# Economic evidence with respect to cost-effectiveness of the transitional care model among geriatric patients discharged from hospital to home: a systematic review

**DOI:** 10.1007/s10198-021-01301-4

**Published:** 2021-04-10

**Authors:** Kristina Kast, Carl-Philipp Wachter, Oliver Schöffski, Martina Rimmele

**Affiliations:** 1 Chair of Health Care Management, Law and Economics Faculty of the Friedrich-Alexander University of Erlangen-Nuremberg, Lange Gasse 20, 90403 Nuremberg, Germany; 2 Medical Faculty of the Friedrich-Alexander University of Erlangen-Nuremberg, Institute for Biomedicine of Aging, Kobergerstr. 60, 90408 Nuremberg, Germany

**Keywords:** Transitional care, Geriatric patients, Hospital discharge, Cost-effectiveness, Economic analysis, Budget impact analysis, I1 Health

## Abstract

**Background:**

The German hospital-to-home discharge management of geriatric patients has long been criticized. The implementation of the American Transitional Care Model (TCM) could help to reduce readmissions and costs. The objective of this review was to check the scientific evidence of the cost-effectiveness of the TCM.

**Methods:**

A systematic literature search in six databases for the time period of 26 years was conducted. The studies had to meet all pre-defined inclusion criteria. The data extraction is based on a criteria chart from literature. The methodological quality was assessed using the tools of the National Heart, Lung, and Blood Institute as well as the Consensus Health Economic Criteria list. The results transferability to German health care system was explained based on the criteria from the literature.

**Results:**

Three American studies met all criteria. They showed partial cost analyses but no full economic analyses. It could be assumed that the economic effect of the TCM changes over time. The costs of a care coordinator could not be determined because few detailed information was reported. The TCM may have negative consequences for hospitals. The results are not transferable to Germany.

**Conclusion:**

There is no scientific evidence for the cost-effectiveness of the defined TCM. The optimal TCM duration still needs to be clarified. A detailed overview with units and prices and an additional consideration of the hospital perspective could help to make the information more transparent when deciding about the TCM implementation. A full economic analysis under German conditions or for similar European countries is necessary.

**Supplementary Information:**

The online version contains supplementary material available at 10.1007/s10198-021-01301-4.

## Introduction and background

About 20 million patients are being discharged from German hospitals every year [1]. One in ten of them requires further outpatient care [2]. The transition of patients from the inpatient to the outpatient setting represents an interruption of the continuity of care that is associated with poor post-discharge outcomes. This problem is especially relevant for geriatric patients because they are exposed to high mental and physical stress after discharge from the hospital. For example, they have more difficulties to cope with everyday life, are affected by longer healing periods, and develop new acute or chronic health problems [3, 4]. This can result in hospital readmissions and causes high costs for the health care system.

Consequently, the German legislator introduced discharge management by law in 2007 [5]. It includes an assessment of risk for poor outcomes shortly before discharge, contacting the relatives of the patient, the execution of the discharge measures, and a brief check of the realization of the execution of the measures at discharge [3, 6]. However, the problems of the interruption of the continuity of care and of the high inpatient costs, caused by readmissions, seem to be unsolved, and the discharge management by law is still being criticized [6]. Considering the prognosis that the proportion of people aged 65 years or older will grow by approximately 20% by 2030 [7], it can be assumed that the problem will exacerbate. An improved solution for the transitional care of geriatric patients in Germany is therefore necessary.

An enhancement of the German discharge management with the components of the American Transitional Care Model (TCM) could be one such solution. The TCM has been developed and evaluated in several studies by Naylor et al. [8]. After that, the core components of the model were summarized by Hirschman et al. [9]. Following this model, a patient to be discharged from a hospital is supported by a qualified permanent contact person for a certain period after discharge who makes regular home visits and is also available by telephone. This person coordinates the entire interdisciplinary and integrated care, involves the relatives, supports the patients to perform their activities of daily living, and increasingly promotes the activation of self-management [8]. Since 2017 (running until 2021) in a project funded by the Federal Joint Committee (the highest decision-making body of care deliverers in Germany) researchers compare the TCM with the German routine care in a randomized controlled trial (RCT) [10].

Depending on the success of the project intervention in terms of its effectiveness and cost-effectiveness, it will be decided whether it will also be implemented in Germany as a reimbursable service of the statutory health insurance funds. In addition to the future project results, the results of previous studies can help decision-makers to make an informed decision. There are already some systematic reviews that examined the effectiveness [11, 12] and costs of different models of transitional care [13–16]. To the best of the authors' knowledge, however, there are no reviews available that address the cost-effectiveness with a narrow focus on the TCM and at the same time on geriatric patients. The objective of this review was therefore to check which scientific evidence already exists concerning the cost-effectiveness of the defined TCM (as planned for Germany).

## Methodology

### Search strategy and databases

A systematic literature search was conducted in databases dealing with both medical and economic issues: PubMed, Science Direct, Scopus, EconBiz, Cochrane Library, and CINAHL. A search term was defined that covered three thematic areas (see supplementary information, Table S1): geriatric, TCM, costs. The operators AND as well as OR were used. The search covered the period from 1 January 1995 to 31 December 2020 and the following filters were used: Search in titles, abstracts, and full texts as well as studies in English or German. The last filter means, that the research studies from other countries were allowed but they had to be written in one of the both languages understandable for the authors and to meet inclusion criteria mentioned below.

### Selection criteria

After the duplicates were removed, the remaining articles were screened independently by two authors. Pre-defined selection criteria were applied to identify citations relevant to the review objective. For inclusion in the review, the subjects of the potentially relevant studies had to be geriatric patients. These are defined as patients at a very high age (80 years or older) or as patients aged 65 or older who also have multiple diseases or at least one chronic disease [4, 17]. The hospitalized patients had to be discharged to home, but not to some other settings like nursing home or palliative care facilities. The readmissions had to be unplanned. The intervention needed to be provided as home visits combined with telephone calls. The care coordination had to be carried out by only one responsible person. Furthermore, the examined intervention had to include at least two additional core components of TCM [9], and should not be finished with discharge. The costs needed to be stated in a quantitative form. If one of the criteria was not met, the respective study was excluded. Articles were also excluded if they had no reference to the topic or were grey literature. The transitional care reviews, however, were checked whether they included studies relevant to the objective of the present work. The differences in screening results were then resolved by discussion of the authors. The process of the literature screening was documented in a PRISMA flow chart as recommended by Moher et al. [18].

### Data extraction and analysis

The data were extracted by one author and checked by another. The contents were extracted using a prepared data collection form based on the recommendations from the literature [19] and included information such as author, objective, study type, setting, economic perspective, key results of the respective studies.

It would be of no value to pool data of different study types because it would lead to false conclusions. This is also not recommended for studies of the same type (here RCTs) if they used different methodological approaches to the economic analysis or different outcomes [20]. For these reasons, it was not possible to perform a meta-analysis in this review, and the extracted data were descriptively analyzed in Excel based on frequencies and, if necessary, own calculations and comparisons.

### Quality assessment

The methodological quality of the included studies was assessed separately regarding the methodology of the clinical and the economic evaluation. For the former, the assessment based on tools for RCTs [21] and for observational studies [22] recommended by the National Heart, Lung, and Blood Institute. These tools contain 14 questions per study type that seems to be an acceptable number compared to other very short or very long checklists [23]. Furthermore, it covers the most important methodological criteria of the respective study types [24, 25]. For the economic part, the Consensus Health Economic Criteria (CHEC) list [26] was used for all studies. This tool is appropriate for the assessment of economic studies carried out in the context of clinical studies and for both full and partial economic analyses [27]. The questions of the respective checklist were answered with "yes", "no" or "unclear". No points were awarded, since according to the literature the scale formation is not considered as an appropriate procedure for valid quality checks [23]. However, to be able to assess the overall result on methodological quality, a reference value of at least 75% of fulfilled criteria of the respective quality assessment instrument was considered high and thus acceptable quality. A criterion was fulfilled if the answer to the question could be clearly “yes”.

### Data presentation and discussion

The results of this review are limited to general characteristics of the studies, patient-related outcomes, resource use, and financial outcomes. Patient-related outcomes are those that are important for an individual patient (e.g. comorbidity-related readmission, satisfaction). The resource use is defined as those outcomes that indicate the consumption of resources in the health care system and are therefore relevant for the statutory health insurance funds (e.g. number of readmissions in total, number of outpatient visits). Financial outcomes include all resource consumptions that are valued in monetary units and stated in quantitative form. The text of the review describes the results starting with the variables that were investigated in all included studies. This is followed by the description of variables that appear in a maximum of two studies and ends with the description of variables examined in a single study. In addition to the results presented in the text, reference is made to the supplementary information at the relevant point if more detailed information is available. The key results of the review are discussed afterwards and their transferability to the German health care system is explained. The transferability assessment is based on criteria recommended by Welte et al. [28]. Compared to other criteria sets [29] this one represents an acceptable number of assessment questions that moreover do not overlap with the criteria of the quality assessment tools used in this review.

## Results

### Literature search

**Fig. 1:**
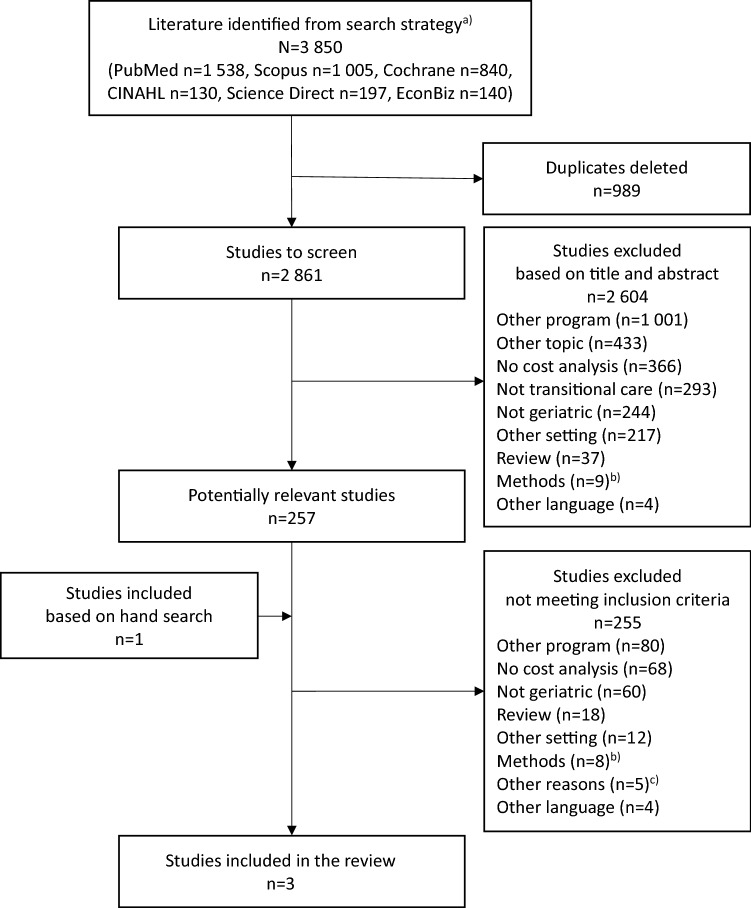
Flow chart for the systematic review process to select studies. ^a^ For more information see online supplementary information, Table S1. ^b^ More than one care coordinator or calls without home visits. ^c^ Economic part of the study mentioned, but not available

The objective of this review was to check which scientific evidence already exists concerning the cost-effectiveness of the defined TCM among geriatric patients. Through the systematic literature search in six databases, a total of 3 850 potentially relevant citations were identified (see Fig. 1). 2 861 of them were screened. Most of the articles (*n* = 2 604) were excluded by screening the titles and abstracts. Further 257 studies had to be screened in full text. In both screening phases, most of the articles (*n* = 1 001 and *n* = 80) were excluded because they were from a different program (e.g. case management, disease management). Other reasons that were often responsible for exclusions was the lack of cost consideration (*n* = 366 and *n* = 68) or addressing other topics (*n* = 433), e.g. flight simulation, dermatological or pharmaceutical issues. In addition, one potentially relevant study was identified through the hand search. Finally, three studies met all criteria and were included in the review: Naylor et al. [30], Naylor et al. [31], and Stauffer et al. [32].

### General characteristics

The general characteristics of the included studies are summarized in Table 1. All three studies were conducted in the USA, two [30, 31] of them by Naylor and colleagues, who designed the TCM. Two studies were RCTs [30, 31], and one an observational study [32]. On average the included studies had 247 subjects while the smallest sample size (*N* = 140) was reported by Stauffer et al. The studies were conducted between 1992 and 2010. The duration of the individual studies ranged from 8 months [32] to almost 4 years [30, 31] with the follow-ups ranging from 2 weeks to 1 year. In all of them, the main focus was on the investigation of the effectiveness of the certain TCM, while the economic consideration was clearly stated as a secondary objective (with an indication of the economic perspective) only in Stauffer et al. and was only generally mentioned in Naylor et al. [31]. All the studies conducted partial cost analyses, but none of them carried out a full economic analysis with a comparison of costs and outcomes. In the quality assessment, none of the studies fulfilled 75% of the quality criteria (for more details see supplementary information, Table S2).

**Table 1:** General characteristics of the studies

Author (year)	Naylor et al. [30]	Naylor et al. [31]	Stauffer et al. [32]
Title	Comprehensive discharge planning and home follow-up of hospitalized elders: A randomized clinical trial	Transitional care of older adults hospitalized with heart failure: A randomized controlled trial	Effectiveness and cost of a transitional care program for heart failure: A prospective study with concurrent controls
Country	USA	USA	USA
Objective(s)	To examine the effectiveness	To examine the sustained effect of the intervention on time to first readmission, […] and medical costs	1) To assess the effectiveness […] in the real-world setting 2) To perform a budget impact analysis for the intervention using costs and reimbursement experience from the intervention
Study type	RCT	Multisite RCT	Before-and-after study with concurrent controls (observational study)
Setting	Hospital of the University of Pennsylvania and the Presbyterian Medical Center of the University of Pennsylvania Health System	Philadelphia academic and community hospitals	Baylor Medical Center Garland […] within the Baylor Health Care System, […] in North Texas
Timeline	August 1992–March 1996	February 1997–January 2001	August 24, 2009–April 30, 2010
Follow-up points	At 2, 6, 12 and 24 weeks	At 2, 6, 12, 26, 52 weeks	At 30, 60 days
Sample^a^	*N* = 363	*N* = 239	*N* = 140
Economic perspective	NR	NR	Budget holder perspective (hospital)
Full economic evaluation	No (description of costs and outcomes)	No (description of costs and outcomes)	No (description of consequences (readmissions, savings))
Sources of funding	National Institute for Nursing Research of the National Institutes of Health	National Institutes of Health funded this study	Supported by the Baylor Health Care System Office of the Chief Quality Officer
Competing interests	NR	NR	NR
Quality of clinical part^b^	< 75%: Yes (7/14); no (2/14); unclear (5/14)	< 75%: Yes (7/14); no (2/14); unclear (5/14)	< 75%: Yes (6/14); no (4/14); unclear (4/14)
Quality of economic part^b^	< 75%: Yes (8/16); no (7/16); unclear (1/16)	< 75%: Yes (8/16); no (7/16); unclear (1/16)	< 75%: Yes (6/16); no (9/16); unclear (1/16)

NR, Not reported

^a^Ø247.333 (= (363 + 239 + 140)/3)

^b^For more information see online supplementary information, Table S2

### Programs and routine care

All three studies fulfilled at least five TCM components (see Table 2). In all programs, the care coordination was carried out by an advanced practice nurse (APN). Furthermore, the components of education, engagement of patients and caregivers, maintaining of relationships, as well as assessment and management of risks and symptoms were fulfilled. The other TCM components were only mentioned in the two RCTs according to Naylor et al. The qualification of the APNs (e.g. in terms of degree, specialization, and experience) was described to different extents in the included studies (e.g. an APN with master´s degree as well as qualification and experience in care coordination of elders [30, 31]). In all of them, the first home visit by APN took place within three days after discharge. The 1-month intervention scheduled at least two home visits [30] and each of the 3-month interventions [31, 32] scheduled at least eight home visits. The APNs were available by telephone 7 days a week.

**Table 2:** Programs and routine care of the studies

Author	Intervention						Control
	Duration	Personnel qualification	Initial home visit	Further visits	APN availability	TCM components	
Naylor et al. [30]	1 month	APN, master´s degree, gerontological specialist, experience in hospital and/or home care for elderly	Within 48 h	Second visit 7–10 days after discharge At least 2 home visits	7 days a week (8 am to 10 pm on weekdays and 8 am to noon on weekends) by telephone	1–9	Discharge planning routine for adult patients at study hospitals If referred, standard home care consistent with Medicare regulations Visiting nurse with bachelor´s degree
Naylor et al. [31]	3 months	APN, master´s degree, general expertise in care conditions of elderly, participated in 2-months program for developing competencies related to recognition and treatment of heart failure in elders	Within 24 h	Weekly home visits during the first month, bimonthly during the second and third months, additional visits based on individual needs Hospital visit within 24 h of initial admission and at least daily At least 8 home visits	7 days per week (8 am to 8 pm weekdays and 8 am to noon weekends) by telephone	1–9	Care routine for the admitting hospital, including site-specific heart failure patient management and discharge planning critical paths If referred, standard home agency care consisting of comprehensive skilled home health services 7 days a week On-call registered nurse availability 24 h per day
Stauffer et al. [32]	3 months	APN	Within 72 h	At least 8 home visits	7 days a week by telephone	2,5,6,8,9	Routine care, including care management assistance with discharge planning and referral for home health care services if appropriate

1 Screening: Targets adults transitioning from hospital to home who are at high risk for poor outcomes

2 Staffing: Uses an advanced practice registered nurse who assume primary responsibility for care management throughout episodes of acute illness

3 Fostering coordination: Promotes communication and connections between healthcare and community-based practitioners

4 Promoting continuity: Prevents breakdowns in care from hospital to home by having same clinician involved across these sites

5 Educating/promoting self-management: Prepares older adults and family caregivers to identify and respond quickly to worsening symptoms

6 Engaging patients and caregivers: Engages older adults in design and implementation of the plan of care aligned with their preferences, values and goals

7 Collaborating: Promotes consensus on plan of care between older adults and members of the care team

8 Maintaining relationships: Establishes and maintains a trusting relationship with the patient and family caregivers involved in the patients’ care

9 Assessing/managing risks and symptoms: Identifies and addresses the patient's priority risk factors and symptoms

The respective interventions were compared with routine care. In the first study it was discharge planning that was routine at the University Hospital of Pennsylvania accomplished by Medicare home care [30]. The routine care of the second study was care at the Philadelphia Academic Hospital with management and discharge planning specifically for heart failure patients, comprehensive skilled home health services seven days a week, and a registered nurse with a telephone availability of 24 h a day [31]. The last study defined the routine care as care management assistance with discharge planning and home health care services [32]. In Naylor et al. [30] a person with a bachelor's degree was the visiting nurse (VN), and in Naylor et al. [31] a registered nurse carried out the routine care.

### Outcomes

#### Patient-related outcomes

All three studies reported that there were no significant differences in mortality between the subjects in the intervention (IG) and control (CG) groups (see Table 3, and Table S3 of the supplementary information for more details on outcomes). According to both RCTs, no significant improvements in functional status were observed, and patient satisfaction either did not improve [30] or was significantly better only in the first 3 months in the IG [31]. Both the number of patients requiring single and multiple readmissions were lower in the IG (significant [31]), and the length of hospital stay per patient was significantly lower in the IG, according to the RCTs. Readmissions related to new health problems were either not significantly higher [31] or only significantly lower at the 10% level [30].

**Table 3:** Results of the studies

Study outcomes^a^	Studies										
	Naylor et al. [30]			Naylor et al. [31]			Stauffer et al. [32]				
	IG (*n* = 177)	CG (*n* = 186)	*p*-value	IG (*n* = 118)	CG (*n* = 121)	*p*-value	TCP patients (*n* = 56)	Nonintervention patients (*n* = 84)	BMCG (*n* = 140)	BHCS (*n* = 885)	*p*-value
Patient-related outcomes											
Patients died	11	11	NR	11	13	0.83	0^b^	NR	2^b^	NR	NR
Patient satisfaction	"No significant group differences"		0.92	"Short-term improvements" in IG^c^		< 0.001	NR	NR	NR	NR	–
Functional status	"No significant group differences"		0.33	"Statistically significant group differences […] did not emerge"		NR	NR	NR	NR	NR	–
Patients needed readmission (at least 1 time)	20.3%^d^	37.1%^d^	< 0.01	44.9%	55.4%	< 0.121	NR	NR	NR	NR	–
Patients needed multiple readmissions (more than 1 time)	6.2%	14.5%	0.01	28.2%	36.4%	< 0.218	NR	NR	NR	NR	–
Time to first readmission for any reason	"Increased" in IG		< 0.001	"Longer" in IG^c^		0.026	NR	NR	NR	NR	–
Index related readmissions	30	64	.005	40	72	< 0.184	NR	NR	NR	NR	–
Comorbidity related readmissions	10	25	0.06	23	50	< 0.013	NR	NR	NR	NR	–
New health problem related readmissions	9	18	0.10	41	40	< 0.881	NR	NR	NR	NR	–
Time in hospital per patient	Ø1.53	Ø4.09	< 0.001	Ø5.0	Ø8.0	< 0.071	NR	NR	NR	NR	–
Time in hospital per readmitted patient	Ø7.5	Ø10.1	< 0.001	Ø11.1	Ø14.5	< 0.411	NR	NR	NR	NR	–
Length of stay for readmitted patients	Ø7.5	11.0	< 0.001	NR	NR	–	NR	NR	NR	NR	–
Time of readmissions (from discharge to 6 weeks	17	47	< 0.001	NR	NR	–	NR	NR	NR	NR	–
Time of readmissions (6 to 24 weeks)	32	60	0.02	NR	NR	-	NR	NR	NR	NR	–
Time of readmissions (from discharge to 30 days)	NR	NR	–	NR	NR	–	Ø8.1	Ø14.1	Ø12.6^e^	Ø16.4^e^	NR
Resource use											
Readmissions, total	49	107	< 0.001	104	162	< 0.047	NR	NR	^f^	^g^	^h^
Acute care visits (unplanned)	Ø1.6 (= 1.5 + 0.1)	Ø1.8 (= 1.6 + 0.2)	–	Ø0.9 (= 0.8 + 0.1)	Ø1.1 (= 0.8 + 0.3)	NR	NR	NR	NR	NR	–
Home visits	Ø14.96 (= 3.1 + 4.5 + 0.03 + 3.5 + 0.1 + 0.03 + 3.7)	Ø14.1 (= 7.1 + 0 + 0.07 + 3.1 + 0.2 + 0 + 3.6)	–	Ø14.8 (= 1.1 + 12.1 + 0.7 + 0 + 0.9)	Ø8.4 (= 6.3 + 0 + 1 + 0 + 1.1)^d^	NR	NR	NR	NR	NR	–
Total visits	Ø16.6	Ø15.9	0.77	Ø15.7 (= 14.8 + 0.8 + 0.1)	Ø9.5^d^	< 0.001^d^	NR	NR	NR	NR	–
Total hospital days	270	760	< 0.001	588	970	0.071	NR	NR	NR	NR	–
Financial outcomes											
Costs per Patient	$3 630	$6 661	< 0.001	$6 152	$9 618	0.002^c^	$6 236^i^	$6 760^i^	NR	NR	–
Total costs	$642 595	$1 238 928	< 0.001	$725 903	$1 163 810	0.404^d^	NR	NR	NR	NR	–
Cost savings, per patient	$3 031 (= 6 661 – 3 630)		–	$3 466 (= 9 618 – 6 152)		–	NR	NR	NR	NR	–
Cost savings, total	$596 333 (= 1 238 928 – 642 595)		–	$437 907 (= 116 3810 – 725 903)		–	NR	NR	NR	NR	–
Costs for visits	$215 378	$214 710	0.72	$138 649	$97 883	<0 .001^d^	NR	NR	NR	NR	–
Costs for home visits	$181 303 (= 101 697 + 79 606)	$176 989 (= 101 049 + 75 940)	NR	$132 321 (= 11 5856 + 16 465)^d^	$87 064 (= 64 531 + 22 533)^d^	NR	NR	NR	NR	NR	–
Direct intervention costs (VN + APN)	$101 697	$101 049	0.72	$115 856^j^	$64 531	NR	NR	NR	NR	NR	–
APN	$61 600	$0	< 0.001	$104 019^j^	$0	–	NR	NR	NR	NR	–
Costs for readmissions	$427 217	$1 024 218	< 0.001	$587 253	$1 065 927	0.088	NR	NR	NR	NR	–
From discharge to 6 months	$427 217	$1 024 218	< 0.001	$381 725	$841 164	0.030	NR	NR	NR	NR	–
From discharge to 3 months	NR	NR	–	$236 144	$489 420	0.10	NR	NR	NR	NR	–
From 6 to 12 months	NR	NR	–	$205 528	$218 035	0.235	NR	NR	NR	NR	–
Program costs	NR	NR	–	NR	NR	–	$1 110^ k^	0	NR	NR	–
Cost savings to hospital	NR	NR	–	NR	NR	–	"Intervention did not save money from hospital perspective"		NR	NR	–
Revenue	NR	NR	–	NR	NR	–	$7 445^e,k^	NR	NR	NR	–
Contribution margin	NR	NR	–	NR	NR	–	$1 209^e,k^		NR	NR	–
Difference in contribution margin per patient	NR	NR	–	NR	NR	–	$-227^ k^	–	NR	NR	^h^

BHCS, Baylor Health Care System; BMCG, Baylor Medical Center Garland; NR, Not reported; TCP, Transitional care program; “- “, Value was not examined or not necessary

^a^For more information see online supplementary information, Table S3

^b^30 days from discharge

^c^("patients satisfaction", Naylor et al. [31]): At 2 and 6 weeks

^c^("time to first readmission", Naylor et al. [31]): Or time to death

^c^(*p*-value 0.002, Naylor et al. [31]): Lin estimate

^d^(Naylor et al. [30]): Contradictory in text and table

^d^(Ø8.4, Naylor et al.[31]): Home visits reported as 9.5, incl. acute care

^d^(Ø9.5, Naylor et al. [31]): Total visits reported as total home visits, but incl. acute care

^d^($132 321, Naylor et al. [31]): Reported as home visits $138 649; incl. acute care

^d^($87 064, Naylor et al. [31]): Reported as home visits $97 883; incl. acute care

^d^(*p*-value 0.404, Naylor et al. 2004): In text reported as "*p* < 0.002"

^d^(*p*-value < 0.001, Naylor et al. 2004): Reported as total home visits

^e^Ø12.6 (before Ø25.2); Ø16.4 (before Ø18.0); $7 445 (before $8 196); $1 209 (before $1 436)

^f^"Adjusted 30-day readmission rate was 48% lower at BMCG after the intervention than before"

^g^”The statistically significant improvement in readmission rates was not observed for the rest of BHCS between the control and post-intervention periods"

^h^The value was “significant”

^i^Average per patient; "Estimate based on 100 patients for cost of index admission plus in-hospital care 30 days after discharge"

^j^APNs incl. "costs of multidisciplinary team members’ services"

^k^Per patient

### Resource use

All three studies reported that readmissions in total were significantly more frequent without the intervention (see Table 3). In both RCTs, the IG subjects spent significantly fewer days in hospital than the CG subjects (270 vs. 760 [30] and 588 vs. 970 [31]). While the average number of total visits and the average number of included home visits was higher in the IGs, both studies reported that the intervention reduced the average number of acute care visits (emergency room, outpatient doctors). According to both studies, on average more APN home visits per patient (see supplementary information, Table S3) were made than scheduled (4.5 vs. 2 [30] and 12.1 vs. 8 [31]). None of the studies provided information on the number of telephone calls made.

### Financial outcomes

All three studies reported that the costs per patient were lower in the IGs. However, only two [30, 31] of them reported a significant effect (see Table 3). In addition, the RCTs showed that the total costs in the IGs were about half of those of the CGs. According to these two studies, this effect could also be shown in relation to the total readmission costs (significant [30]). After half a year of follow-up both RCTs had significantly lower readmission costs in the IGs. In addition, both reported that the direct program costs (defined as visits by APNs and VNs) in the IGs were just over $100 000. The CG in Naylor et al. [30] showed slightly lower costs than the IG, while the CG by Naylor et al. [31] was half as expensive as the IG. According to these two studies, the total costs for all visits—and explicitly for home visits (including other service providers such as physiotherapists)—were always higher in the IGs of the respective study. The costs of APNs were $61 600 after 1-month intervention in Naylor et al. [30], and were almost twice as high after the 3-month intervention in the Naylor et al. [31] study. Cost savings were reported in both RCTs. Naylor et al. [30] reported $596 333 in total and $3 031 per patient, and Naylor et al. [31] reported $437 907 in total and $3 466 per patient (despite more expensive APNs and lower costs for acute care visits). Only Stauffer et al. reported program costs as $1 110 per patient considering the perspective of the hospital as the budget holder. According to this study, the program did not save the money from the hospital perspective, but the hospital recorded a loss of contribution margin of $227 per patient over 30 days, which was considered “significant” [32].

## Discussion

### Patient-related outcomes and resource use

With regard to patient-related outcomes, the included studies reported that there were no differences in mortality [30–32], and that the programs led to significantly shorter hospital length of stay and significantly longer time to first readmission [30, 31]. No tendencies are discernible in other outcomes, as these were investigated either in two studies with different results (e.g. satisfaction) or in only one study (e.g. quality of life). Regarding the resource use, it was found that readmissions were about half as often at a significant level [30–32], and hospital days were reduced by one to two thirds [30, 31]. No significant difference in the number of outpatient resources (total visits) was reported in one study [30], while another showed a significant increase by one-third [31]. Since the resource use in the latter study corresponds to the sum of the total visits but is reported as home visits by the service providers, the effect size and the associated p-value cannot be assumed with certainty. There were no differences in the use of acute care visits by service providers, but there were differences in home visits by the nurses. Since this value strongly depends on the minimum visits scheduled for the respective program, it cannot be regarded as the actual number of visits required. Furthermore, all data concerning patient-related outcomes and resource use referred to different time periods. For these reasons, no final conclusive statements regarding these types of results can be made in this review. Overall, the studies included in the review provide isolated indications that improvements regarding some patient-related outcomes as well as reductions in the inpatient resource use are possible. However, based on the included studies it remains unclear which increase in resources in the outpatient sector can be expected concretely, and with which intensity the single resources (e.g. home visits, telephone calls) should be used. For a valid assessment of patient-related outcomes and resource use, further studies should have been considered, but these had to be excluded due to the lack of cost consideration.

### Financial outcomes

Both RCTs reported that the total readmission costs in the IGs were significantly lower after 6 months of follow-up (*p* < 0.001 [30] and *p* = 0.030 [31]). Considering that the intervention duration was 1 month in Naylor et al. [30], and the readmission costs of the CG were 2.40 times higher in relation to the IG—while they were only 2.21 times higher with the intervention duration of 3 months in Naylor et al. [31]—it can be assumed that the economic effect of the TCM changes over time. In Naylor et al. [31] the total readmission costs in the CG were 2.07 times higher after 3-month follow-up (*p* = 0.010), and only 1.06 times higher for the period from 6 months to 1 year (*p* = 0.235) of follow-up. This result would support the assumption. A similar effect in terms of total costs per patient can be observed: While the costs in Stauffer et al. were 1.08 times higher in the CG after intervention and follow-up duration of 1 month, they were 1.83 times higher after 1-month intervention and 6 months of follow-up in Naylor et al. [30], and only 1.56 times higher in Naylor et al. [31] with 3-month intervention and 1 year of follow-up. Furthermore, the total savings in the study with longer intervention duration and follow-up [31] appear to be lower than after half a year with one-month intervention ($437 907 [31] vs. $596 333 [30]). Although this effect is contrary per patient ($3 466 [31] vs. $3 031 [30]), this is an additional amount of only $435. Therefore, it is necessary to further investigate which duration is optimal for the TCM to be able to achieve the best economic results.

In addition, both RCTs reported that the direct program costs—defined as visits by APNs and VNs—in each IG amounted to just over $100 000. More precisely, $61 600 was spent on APNs for 1 month of TCM [30] and $104 019 for 3 months [31]. Stauffer et al. reported the average monthly program costs of $1 110 per patient. Converted to 1 month the APN costs per patient would thus vary heavily (approximately between $300 and $1 000). The reason for this could be the lack of details in the cost composition. Stauffer et al., for example, did not give any concrete information on the components of the program costs, and Naylor et al. [31] stated that the APN costs also included the costs for the multidisciplinary team (without exact amount). To be able to make more reliable statements regarding the expected costs for a care coordinator a separate list of detailed quantities (e.g. working hours or days) and prices (e.g. salaries) must be available.

Finally, Stauffer et al. calculated in a Budget Impact Analysis (BIA) that the study hospital had a loss of contribution margin. This contradicts the positive economic effects (e.g. savings) emphasized in the other two studies [30, 31], but is not unexpected as this analysis took a different perspective [33]. Not enough detailed information is available to fully assess the quality of the performed BIA. However, apart from the limitations in the methodological quality of the study mentioned above (Table 2) it must be considered that the BIA was estimated for a relatively short period of time on a small sample. The selected time horizon of only 1 month with an intervention duration of 3 months as well as a selective sample of 100 patients should be justified in detail. In addition, the estimated costs were based on the prices/reimbursement amounts of the respective American budget holder, and since no separate unit data were reported, the significance for other budget holders (e.g. in Germany) cannot be estimated [34]. In general, however, it seems plausible that the reduction of readmissions by the program could have negative economic consequences for hospitals since in particular geriatric patients in higher age and with certain diagnoses can generate a considerable share of hospital revenues [35]. In addition to a comparison of costs and effectiveness in a full economic analysis, an additional BIA based on the reimbursement amounts of the respective budget holder with subgroup analyses according to age and diagnosis can be useful [33] to gain the most transparent information for decision-makers in the health care system.

### Transferability to German health care system

According to Welte et al. [28] the following three main criteria have to be fulfilled in order to be able to make concrete statements based on the results of included studies with regard to Germany: The interventions and comparators have to be as similar as possible, and the studies have to be of acceptable quality. Due to the restrictive selection criteria, the three included interventions are very similar to the intervention planned for Germany. However, the comparators of the CGs of the studies differ from the German routine care. It can be seen, for example, that the 24-h availability of the contact person seven days a week in Naylor et al. [31] does not correspond to German routine care. Other studies provide less concrete information by talking about routine care according to the standards of the respective study hospitals (e.g. home visits by VN). Regarding the criterion of acceptable methodological quality, the included studies could not be classified as internally valid, since the bias could not be completely excluded (see Table 1 and supplementary information, Table S2). In the evaluation of the economic part, it was shown that the proportion of questions in the CHEC list clearly answered with "yes" was clearly below 75% for all three studies. The clinical part, which is an important basis for the economic analysis, was also fulfilled in exactly half of the criteria in each of the RCTs. In the observational study, which is assigned a lower evidence level in the literature compared to the RCTs [36, 37], only 6 of 14 criteria were fulfilled. The results transferability to Germany is not given for these reasons. Therefore, a full economic analysis under German conditions or in the context of other European countries with similar health care [38] and reimbursement systems [39] is important.

## Limitations

The present review is based on very restrictive selection criteria for identified studies. Although this was necessary to reflect the planned intervention and the affected patient population as closely as possible, it also led to the exclusion of very similar studies (e.g. [40, 41]). In the assessment of the methodological quality the included studies were also strictly evaluated, as the border of 75% was considered as an acceptable quality, and this could only be reached by criteria clearly classified as “yes". In addition, the choice of the quality assessment instrument may also have played a role. Researchers may come to other conclusions regarding the methodological quality of the included studies when using other instruments [42]. Nevertheless, it would not change the conclusion regarding the transferability of the results since the criterion of similar routine care was not fulfilled. Furthermore, the included studies differed in study type, duration of intervention, and follow-up, examined few common outcomes, and reported few detailed information. It was therefore not possible to combine the data to perform own cost-effectiveness calculations, and only selected outcomes could be compared and discussed.

## Conclusion

There is no scientific evidence for the cost-effectiveness of the TCM defined in this review, but three studies with partial cost analyses could be identified. The analysis of the included partial cost considerations indicates that it still needs to be clarified which duration is optimal for the TCM to achieve the best economic results. Furthermore, it was found that a detailed overview with units and prices is necessary to determine the care coordinator costs and that additional consideration of the hospital perspective could make information more transparent, and could help to make an informed decision regarding the implementation of the TCM. In any case, a separate full economic analysis under German conditions or for similar European countries is necessary.

## Supplementary Information

Below is the link to the electronic supplementary material.

Supplementary file 1 (PDF 217 KB)

## Data Availability

The datasets used and/or analyzed during the current study are available from the corresponding author on reasonable request.

## Abbreviations

APN: Advanced practice nurse
BIA: Budget impact analysis
BHCS: Baylor Health Care System
BMCG: Baylor Medical Center Garland
CG: Control group
IG: Intervention group
NR: Not reported
RCT: Randomized controlled trial
TCM: Transitional care model
TCP: Transitional care program
VN: Visiting nurse
