# Supplementary material for: Economic evidence with respect to cost-effectiveness of the transitional care model among geriatric patients discharged from hospital to home: a systematic review
Source: Eur J Health Econ. 2021 Apr 10;22(6):961–75. doi: 10.1007/s10198-021-01301-4 (PMC8275561; doi:10.1007/s10198-021-01301-4)
Supplement: Supplementary file 1 — (PDF 217 KB) [file 10198_2021_1301_MOESM1_ESM.pdf]

Economic evidence of the transitional care model among geriatric patients discharged from hospital to home: A systematic review

Supplementary information

Content

Table S1 Final search strategy 2

Table S2 Methodological quality assessment 3

Table S3 Results of the studies.. 6

Table S1 Final search strategy

(geriatric OR elderly OR chronic* OR multimorbid OR “older people” OR frail OR vulnerab* OR “complex care needs”)
AND
(TCM OR “transitional care” OR “transition of care” OR “transitional follow-up” OR “transitional case” OR “transitional discharge” OR “care transition” OR “transitional program” OR “health-social transitional care” OR “hospital-based discharge transition program” OR “postdischarge care transition” OR “transition coach” OR “transitional care pathways” OR “transitional care protocols” OR transsectoral OR “post-discharge support” OR “telephone calls support” OR “home visits” OR “home-based care” OR “discharge to home” OR “post-discharge” OR “self-management” OR “needs-oriented discharge planning” OR “discharge planning” OR “nurse practitioner management” OR “nurse case management” OR “health service intervention”)
AND
(“cost-savings” OR efficien* OR “cost-effective” OR “cost-effectiveness” OR “cost-benefit” OR “cost-utility” OR “cost analysis” OR “cost perspective” OR incremental OR “economic evaluation” OR “economic effect” OR “health economic study” OR “life-quality” OR “quality-adjusted life years” OR “quality-adjusted life-years” OR QALY OR readmission OR rehospitalization OR “hospital length of stay” OR “bed-days”)

Table S2 Methodological quality assessment

Area	Studies		
	Naylor et al. (1999)	Naylor et al. (2004)	Stauffer et al. (2011)
**Clinical part**			
RCTs			
1. Was the study described as randomized, a randomized trial, a randomized clinical trial, or an RCT?	Yes	Yes	-
2. Was the method of randomization adequate (i.e., use of randomly generated assignment)?	Yes	Yes	-
3. Was the treatment allocation concealed (so that assignments could not be predicted)?	Unclear	Unclear	-
4. Were study participants and providers blinded to treatment group assignment?	Yes	Yes	-
5. Were the people assessing the outcomes blinded to the participants' group assignments?	Unclear	Unclear	-
6. Were the groups similar at baseline on important characteristics that could affect outcomes (e.g., demographics, risk factors, co-morbid conditions)?	Yes	Yes	-
7. Was the overall drop-out rate from the study at endpoint 20% or lower of the number allocated to treatment?	No	No	-
8. Was the differential drop-out rate (between treatment groups) at endpoint 15 percentage points or lower?	No	Yes	-
9. Was there high adherence to the intervention protocols for each treatment group?	Unclear	Unclear	-
10. Were other interventions avoided or similar in the groups (e.g., similar background treatments)?	Unclear	Unclear	-
11. Were outcomes assessed using valid and reliable measures, implemented consistently across all study participants?	Unclear	Unclear	-
12. Did the authors report that the sample size was sufficiently large to be able to detect a difference in the main outcome between groups with at least 80% power?	Yes	Yes	-
13. Were outcomes reported or subgroups analyzed pre-specified (i.e., identified before analyses were conducted)?	Yes	No	-
14. Were all randomized participants analyzed in the group to which they were originally assigned, i.e., did they use an intention-to-treat analysis?	Yes	Yes	-
Overall assessment	(<75 %)	<75 %	-
Observational study			
1. Was the research question or objective in this paper clearly stated?	-	-	Yes
2. Was the study population clearly specified and defined?	-	-	Yes
3. Was the participation rate of eligible persons at least 50%?	-	-	No
4. Were all the subjects selected or recruited from the same or similar populations (including the same time period)? Were inclusion and exclusion criteria for being in the study pre-specified and applied uniformly to all participants?	-	-	No

5. Was a sample size justification, power description, or variance and effect estimates provided?	-	-	No
6. For the analyses in this paper, were the exposure(s) of interest measured prior to the outcome(s) being measured?	-	-	Yes
7. Was the timeframe sufficient so that one could reasonably expect to see an association between exposure and outcome if it existed?	-	-	Yes
8. For exposures that can vary in amount or level, did the study examine different levels of the exposure as related to the outcome (e.g., categories of exposure, or exposure measured as continuous variable)?	-	-	No
9. Were the exposure measures (independent variables) clearly defined, valid, reliable, and implemented consistently across all study participants?	-	-	Unclear
10. Was the exposure(s) assessed more than once over time?	-	-	Unclear
11. Were the outcome measures (dependent variables) clearly defined, valid, reliable, and implemented consistently across all study participants?	-	-	Yes
12. Were the outcome assessors blinded to the exposure status of participants?	-	-	Unclear
13. Was loss to follow-up after baseline 20% or less?	-	-	Unclear
14. Were key potential confounding variables measured and adjusted statistically for their impact on the relationship between exposure(s) and outcome(s)?	-	-	Yes
Overall assessment	-	-	<75 %
**Economic part**			
Is the study population clearly described?	Yes	Yes	No
Are competing alternatives clearly described?	No	Yes	No
Is a well-defined research question posed in answerable form?	No	No	No
Is the economic study design appropriate to the stated objective?	No	No	No
Is the chosen time horizon appropriate to include relevant costs and consequences?	Yes	Yes	Yes
Is the actual perspective chosen appropriate?	No	No	Yes
Are all important and relevant costs for each alternative identified?	Yes	Yes	Yes
Are all costs measured appropriately in physical units?	Unclear	Unclear	No
Are costs valued appropriately?	Yes	Yes	Unclear
Are all important and relevant outcomes for each alternative identified?	No	No	No
Are all outcomes measured appropriately?	No	No	Yes
Are outcomes valued appropriately?	Yes	Yes	Yes
Is an incremental analysis of costs and outcomes of alternatives performed?	-	-	-
Are all future costs and outcomes discounted appropriately?	-	-	-
Are all important variables, whose values are uncertain, appropriately subjected to sensitivity analysis?	Yes	No	No

Do the conclusions follow from the data reported?	Yes	Yes	No
Does the study discuss the generalizability of the results to other settings and patient/client groups?	Yes	No	Yes
Does the article indicate that there is no potential conflict of interest of study researcher(s) and funder(s)?	No	No	No
Are ethical and distributional issues discussed appropriately?	-	-	-
Overall assessment	<75 %	<75 %	<75 %

Table S3 Results of the studies

Study outcomes	Studies										
	Naylor et al. (1999)			Naylor et al. (2004)			Stauffer et al. (2011)				
	IG (n=177)	CG (n=186)	p-value	IG (n=118)	CG (n=121)	p-value	TCP patients (n=56)	Nonintervention patients (n=84)	BMCG (n=140)	BHCS (n=885)	p-value
Patient related outcomes											
Patient satisfaction	"no significant group differences"		.92	"short-term improvements" in IG *		<.001	NR	NR	NR	NR	-
Functional status	"no significant group differences"		.33	"statistically significant group differences […] did not emerge"		NR	NR	NR	NR	NR	-
Patients needed readmission (at least 1 time)	20.3 % !	37.1 % !	<.01	44.9 %	55.4 %	<.121	NR	NR	NR	NR	-
Patients needed multiple readmissions (more than 1 time)	6.2 %	14.5 %	.01	28.2 %	36.4 %	<.218	NR	NR	NR	NR	-
Time to first readmission for any reason	"increased" in IG		<.001	"longer" in IG *		.026	NR	NR	NR	NR	-
Index related readmissions	30	64	.005	40	72	<.184	NR	NR	NR	NR	-
Comorbidity related readmissions	10	25	.06	23	50	<.013	NR	NR	NR	NR	-
New health problem related readmissions	9	18	.10	41	40	<.881	NR	NR	NR	NR	-
Time in hospital per patient	Ø 1.53	Ø 4.09	<.001	Ø 5.0	Ø 8.0	<.071	NR	NR	NR	NR	-
Time in hospital per readmitted patient	Ø 7.5	Ø 10.1	<.001	Ø 11.1	Ø 14.5	<.411	NR	NR	NR	NR	-
Depression	"no significant group differences"		.20	NR	NR	-	NR	NR	NR	NR	-
Readmissions per patient, per year	NR	NR	-	Ø 1.18	Ø 1.79	<.001	NR	NR	NR	NR	-
Patients died	11	11	NR	11	13	.83	0 #	NR	2 #	NR	NR

Quality of life	NR	NR	-	"short-term improvements" in IG *		<.05	NR	NR	NR	NR	-
Length of stay for readmitted patients	Ø 7.5	Ø 11.0	<.001	NR	NR	-	NR	NR	NR	NR	-
Time of readmissions (from discharge to 30 days)	NR	NR	-	NR	NR	-	Ø 8.1	Ø 14.1	Ø 12.6 §	16.4 Ø §	NR
Time of readmissions (from discharge to 6 weeks)	17	47	<.001	NR	NR	-	NR	NR	NR	NR	-
Time of readmissions (6 to 24 weeks)	32	60	.02	NR	NR	-	NR	NR	NR	NR	-
Time of readmissions (from discharge to 3 months)	NR	NR	-	NR	NR	-	NR	NR	NR	NR	-
Time of readmissions (from discharge to 6 months)	NR	NR	-	NR	NR	-	NR	NR	NR	NR	-
Time of readmissions (6 months to 1 year)	NR	NR	-	NR	NR	-	NR	NR	NR	NR	-
Resource use											
Readmissions, total	49	107	<.001	104	162	<.047	NR	NR	"Adjusted 30-day readmission rate was 48% lower at BMCG after the intervention than before"	"The statistically significant improvement in readmission rates was not observed for the rest of BHCS between the control and post-intervention periods"	"significant"
Total hospital days	270	760	<.001	588	970	.071	NR	NR	NR	NR	-

Total visits	Ø 16.6	Ø 15.9	.77	Ø 15.7 (=14.8+0.8+0.1)	Ø 9.5 !	<.001 !	NR	NR	NR	NR	-
Acute care visits (unplanned)	Ø 1.6 (=1.5+0.1)	Ø 1.8 (=1.6+0.2)	-	Ø 0.9 (=0.8+0.1)	Ø 1.1 (=0.8+0.3)	NR	NR	NR	NR	NR	-
Physician	Ø 1.5	Ø 1.6	.59	Ø 0.8	Ø 0.8	.609	NR	NR	NR	NR	-
Emergency	Ø 0.1	Ø 0.2	.21	Ø 0.1	Ø 0.3	.116	NR	NR	NR	NR	-
Home visits	Ø 15 (=3.1+4.5+0.03+ 3.5+0.1+0.03+3.7)	Ø 14.1 (=7.1+0+0.07+ 3.1+0.2+0+3.6)	-	Ø 14.8 (=1.1+12.1+0.7+ 0+0.9)	Ø 8.4 (=6.3+0+1+0 +1.1) !	NR	NR	NR	NR	NR	-
VN	Ø 3.1	Ø 7.1	.05	Ø 1.1	Ø 6.3	<.001	NR	NR	NR	NR	-
APN	Ø 4.5	Ø 0	<.001	Ø 12.1	Ø 0	-	NR	NR	NR	NR	-
Social worker	Ø 0.03	Ø 0.07	.23	Ø 0	Ø 0	.678	NR	NR	NR	NR	-
Physical therapists	Ø 3.5	Ø 3.1	.32	Ø 0.7	Ø 1.0	.703	NR	NR	NR	NR	-
Occupational ther-apists	Ø 0.1	Ø 0.2	.95	NR	NR	-	NR	NR	NR	NR	-
Speech therapists	Ø 0.03	Ø 0	.31	NR	NR	-	NR	NR	NR	NR	-
Home health aids	Ø 3.7	Ø 3.6	.46	Ø 0.9	Ø 1.1	.286	NR	NR	NR	NR	-
Total calls	NR	NR	-	NR	NR	NR	NR	NR	NR	NR	-
Length of stay	NR	NR	-	NR	NR	-	NR	NR	"little difference […] between MBCG and BHCS"		"[…] the in-tervention did not have a significant effect"
Financial outcomes											
Costs per Patient	$ 3 630	$ 6 661	<.001	$ 6 152	$ 9 618	.002 *	$ 6 236 ##	$ 6 760 ##	NR	NR	-
Total costs	$ 642 595	$ 1 238 928	<.001	$ 725 903	$ 1 163 810	.404 !	NR	NR	NR	NR	-
Cost savings, per patient	$ 3 031 (=6 661-3 630)		-	$ 3 466 (=9 618-6 152)		-	NR	NR	NR	NR	-
Cost savings, total	$ 596 333 (=1 238 928-642 595)		-	$ 437 907 (=1 163 810-725 903)		-	NR	NR	NR	NR	-
Costs for visits	$ 215 378	$ 214 710	.72	$ 138 649	$ 97 883	<.001 !	NR	NR	NR	NR	-
Costs for acute care visits	$ 34 075	$ 37 721	.74	$ 6 329	$ 10 819	NR	NR	NR	NR	NR	-

Physicians	$ 24 937	$ 27 121	.82	$ 4 549	$ 5 169	.636	NR	NR	NR	NR	-
Emergency	$ 9 138	$ 10 600	.78	$ 1 780	$ 5 650	.105	NR	NR	NR	NR	-
Costs for home visits	$ 181 303 (=101 697+79 606)	$ 176 989 (=101 049+75 940)	NR	$ 132 321 (=11 5856+16 465) !	$ 87 064 (=64 531+22 533) !	NR	NR	NR	NR	NR	-
Nurses	$ 101 697	$ 101 049	.72	$ 115 856 &	$ 64 531	NR	NR	NR	NR	NR	-
VN	$ 40 097	$ 101 049	.05	$ 11 837	$64 531	<.001	NR	NR	NR	NR	-
APN	$ 61 600	$ 0	<.001	$ 104 019 &	$ 0	-	NR	NR	NR	NR	-
Direct intervention costs (VN+APN)	$ 101 697	$ 101 049	.72	$ 115 856 &	$ 64 531	NR	NR	NR	NR	NR	-
Costs for readmissions	$ 427 217	$ 1 024 218	<.001	$ 587 253	$ 1 065 927	.088	NR	NR	NR	NR	-
From discharge to 3 months	NR	NR	-	$ 236 144	$ 489 420	.10	NR	NR	NR	NR	-
From discharge to 6 months	$ 427 217	$ 1 024 218	<.001	$ 381 725	$ 841 164	.030	NR	NR	NR	NR	-
From 6 to 12 months	NR	NR	-	$ 205 528	$ 218 035	.235	NR	NR	NR	NR	-
Other visits	$ 79 606	$ 75 940	.70	$ 16 465 (=7 120+178+9 167)	$ 22 533 (=10 918+534+11 081)	NR	NR	NR	NR	NR	-
Physical therapists	$ 44 819	$ 40 803	.82	$ 7 120	$ 10 918	.708	NR	NR	NR	NR	-
Occupational therapists	$ 912	$ 2 722	.33	NR	NR	-	NR	NR	NR	NR	-
Speech therapists	$ 474	$ 0	.32	NR	NR	-	NR	NR	NR	NR	-
Social worker	$ 676	$ 1 252	.40	$ 178	$ 534	.678	NR	NR	NR	NR	-
Home health aids	$ 32 725	$ 31 163	.78	$ 9 167	$ 11 081	.286	NR	NR	NR	NR	-
Direct inpatient costs, 60 days from index admission	NR	NR	-	NR	NR	-	Ø $ 4 630	Ø $ 5 200	Ø $ 5 176 §	Ø $ 6 107 §	NR
Program costs	NR	NR	-	NR	NR	-	$ 1 110 **	0	NR	NR	-
Cost savings to hospital	NR	NR	-	NR	NR	-	"intervention did not save money from hospital perspective"		NR	NR	-

Revenue	NR	NR	-	NR	NR	-	$ 7 445 § **	NR	NR	NR	-
Contribution mar-gin	NR	NR	-	NR	NR	-	$ 1 209 § **		NR	NR	-
Difference in con-tribution margin per patient	NR	NR	-	NR	NR	-	$ -227 **		NR	NR	"significant"

IG=Intervention group; CG=Control group; TCP=Transitional care program; BMCG=Baylor Medical Center Garland; BHCS=Baylor Health Care System; NR=Not reported; APN=Advanced practice nurse; VN=Visiting nurse; “-“=Value was not examined or not necessary

30 days from discharge

Average per patient; "Estimate based on 100 patients for cost of index admission plus in-hospital care 30 days after discharge"

* ("patients satisfaction", Naylor et al. 2004): At 2 and 6 weeks
* ("time to first readmission", Naylor et al. 2004): Or time to death
* ("quality of life”, Naylor et al. 2004): At 12 weeks
* (p-value .002, Naylor et al. 2004): Lin estimate

** Per patient

! (Naylor et al. 1999): Contradictory in text and table
! (Ø 8.4, Naylor et al. 2004): Home visits reported as 9.5, incl. acute care
! (Ø 9.5, Naylor et al. 2004): Total visits reported as total home visits, but incl. acute care
! ($ 132 321, Naylor et al. 2004): Reported as home visits $ 138 649; incl. acute care
! ($ 87 064, Naylor et al. 2004): Reported as home visits 97 883 $; incl. acute care
! (p-value .404, Naylor et al. 2004): In text reported as "p<.002"
! (p value <.001, Naylor et al. 2004): Reported as total home visits
§ Ø 12.6 (before Ø 25.2); Ø 16.4 (before Ø 18.0); Ø $ 5 176 (before Ø $ 5 729); Ø $ 6 107 (before Ø $ 6 980); $ 7 445 (before $ 8 196); $ 1 209 (before $ 1 436)
& APNs incl. "costs of multidisciplinary team members’ services"
